# Temperature Sensitivity of Microbial Respiration of Fine Root Litter in a Temperate Broad-Leaved Forest

**DOI:** 10.1371/journal.pone.0117694

**Published:** 2015-02-06

**Authors:** Naoki Makita, Ayumi Kawamura

**Affiliations:** 1 Kansai Research Center, Forestry and Forest Products Research Institute, Kyoto 612-0855, Japan; 2 Graduate School of Agriculture, Kyoto University, Kyoto 606-8502, Japan; National Taiwan University, TAIWAN

## Abstract

The microbial decomposition respiration of plant litter generates a major CO_2_ efflux from terrestrial ecosystems that plays a critical role in the regulation of carbon cycling on regional and global scales. However, the respiration from root litter decomposition and its sensitivity to temperature changes are unclear in current models of carbon turnover in forest soils. Thus, we examined seasonal changes in the temperature sensitivity and decomposition rates of fine root litter of two diameter classes (0–0.5 and 0.5–2.0 mm) of *Quercus serrata* and *Ilex pedunculosa* in a deciduous broad-leaved forest. During the study period, fine root litter of both diameter classes and species decreased approximately exponentially over time. The *Q*_10_ values of microbial respiration rates of root litter for the two classes were 1.59–3.31 and 1.28–6.27 for *Q. serrata* and 1.36–6.31 and 1.65–5.86 for *I. pedunculosa*. A significant difference in *Q*_10_ was observed between the diameter classes, indicating that root diameter represents the initial substrate quality, which may determine the magnitude of *Q*_10_ value of microbial respiration. Changes in these *Q*_10_ values were related to seasonal soil temperature patterns; the values were higher in winter than in summer. Moreover, seasonal variations in *Q*_10_ were larger during the 2-year decomposition period than the 1-year period. These results showed that the *Q*_10_ values of fine root litter of 0–0.5 and 0.5–2.0 mm have been shown to increase with lower temperatures and with the higher recalcitrance pool of the decomposed substrate during 2 years of decomposition. Thus, the temperature sensitivity of microbial respiration in root litter showed distinct patterns according to the decay period and season because of the temperature acclimation and adaptation of the microbial decomposer communities in root litter.

## Introduction

Soil respiration in terrestrial ecosystems is critical for assessing the net ecosystem carbon (C) balance because it represents the second largest global C flux (68–80 PgC/year) between ecosystems and the atmosphere [1]. This amount is over 10 times of that currently produced by fossil fuel combustion. Thus, even a small change in soil respiration could significantly has impacts on the current changes in atmospheric CO_2._ Rising atmospheric CO_2_ enhances greenhouse effects, likely resulting in global warming. The global warming could substantially stimulate soil respiration, resulting in more release of CO_2_ to the atmosphere. Consequently, there is a positive feedback loop between the climatic system and global C cycle [2]. Therefore, increasing efforts have been made to develop a mechanistic understanding of how temperature and other environmental factors affect soil respiration [3].

An important factor that influences the response of soil respiration to global change and climate warming is temperature sensitivity [4], which is often modeled with the *Q*_10_ temperature coefficient, i.e., the rate of change in a process because of increasing the temperature by 10°C. Experimental studies of *Q*_10_ related to soil respiration have demonstrated that the *Q*_10_ values vary with temperature and soil moisture [4, 5]. It has long been recognized that soil respiration is sensitive to temperature, but many questions remain unanswered about the dynamic responses of autotrophic and heterotrophic respiration to temperature changes. In theory, soil respiration is the sum of the autotrophic component produced by roots and the heterotrophic component derived from soil microorganisms that decompose organic materials in litter. Several studies showed that root respiration may be more sensitive to temperature change than microbial respiration [6, 7]. Thus, the temperature dependency of soil respiration varies among different components [4, 8].

Microbial respiration derives from the decomposition and mineralization of C from litter residues by microbial activity. The microbial respiration from soils has been estimated at 53–57 Pg C yr^−1^ globally and is typically 30–80% of total annual soil respiration regulating by environmental factors such as temperature and moisture and biotic factors [9, 10]. Sultzman et al. [11] reported that aboveground litter decomposition contributed 19%, and belowground litter decomposition contributed 58% to total soil CO_2_ efflux in an old growth coniferous forest located in Oregon. An increasing evidence suggests that *Q*_10_ of the respiration varies with the quantity and quality of soil organic matter (SOM) [12–14]. Liski et al. [15] reported that the temperature sensitivity of decomposition was lower for recalcitrant SOM than labile SOM on the basis of soil C storage, suggesting that old SOM is less sensitive to temperature changes. In contrast, Conant et al. [13] suggested that the decomposition of more recalcitrant SOM yields a higher *Q*_10_. Thus, the results of analyses for the temperature sensitivity of microbial respiration remain controversial [16].

Furthermore, despite the large number of studies of the temperature sensitivity of decomposition, more studies of this type are required to understand the temperature sensitivity of litter fractions at different decomposition stages. Few studies have examined how litter quality is related to the temperature responses of microorganisms, and the temperature sensitivity of leaf litter decomposition during the early stage has only rarely been measured [17, 18]. Comparative root litter studies have seldom been conducted in natural forests because of the difficulty of measuring dead root litter. Therefore, it seems absolute values for *Q*_10_ of the root litters cannot be compared among studies. These knowledge gaps limit our ability to predict how global warming will affect the C cycling in forest ecosystems through changes in the temperature sensitivity.

Tree fine roots (<2 mm) are dynamic and short-lived, supplying considerable belowground litter inputs and accounting for up to 75% of the net primary production of forest ecosystems [19]. After root death, the decomposition of fine roots represents an important heterotrophic source of CO_2_ [20, 21]. During root decomposition, litter quality may vary because of the ingrowth of fungal and bacterial cells in fresh litter and the transfer of mineral nutrients as litter decay progresses [22]. Across a wide range of concentrations, mass loss is directly proportional to litter N and lignin and inversely proportional to C/N and lignin/N ratios [23, 24]. However, the microbial respiration derived from early-stage root litters in forests remains ambiguous [21]. In addition, little information is available about continuous changes in the relationship between the temperature sensitivity of microbial respiration and litter quality in root litter. Thus, to improve our understanding and ability to predict quantitative contribution of fine root decomposition to microbial and soil respiration in forest ecosystems, it is necessary to determine the absolute values of temperature sensitivity of respiration in fine root litter relative to different substrate qualities and decay periods.

The study aimed to elucidate the temperature sensitivity of fine root litter throughout the microbial decomposition of fine root litter over several seasons and decay periods. We measured the *Q*_10_ values for microbial respiration in dead litter of two fine root diameter classes (0–0.5 and 0.5–2.0 mm) of *Quercus serrata* and *Ilex pedunculosa*, which are the dominant tree species in broad-leaved forests in Japan. Recent studies reported that fine root systems comprise individual roots with heterogeneous physiological and chemical properties through different root development [25, 26]. Moreover, the substantial heterogeneity among diameter classes in fine roots may influence the direction of effects on litter quality, as well as subsequent impacts on litter decomposition and soil C sequestration. In fine roots, the decomposition rate of smaller roots is slower than that of larger roots, even among <2 mm diameter classes, and their factors controlling the microbial degradation are different because of their different chemical and morphological properties [27–29]. We clearly need more evidence for validating the patterns of these decomposition processes within fine root litters. We here tested the hypothesis that the temperature sensitivity of microbial respiration in root litter differs with the decay period and season because of differences in (1) the seasonal acclimation of microorganisms and (2) decomposition processes in different qualities of litter among species and diameter classes.

## Methods

### Ethics statement

The study site (Yamashiro Experimental Forest) is maintained by the Forestry and Forest Products Research Institute. All necessary permits were obtained for the field study, and the study did not involve endangered or protected species.

### Study site

This study was conducted at the Yamashiro Experimental Forest, Kyoto, which is in a mountainous region of western Japan (34°47’ N, 135°50’ E; 180–250 m above sea level). The mean annual precipitation was 1449 mm, and the mean annual temperature was 15.5°C. Soil was regosol with sandy loam or loamy sand texture and contained fine granite gravel. The mean soil depth at the site was 50 cm, and the soils below 50 cm were classified into the C horizon [26], which contains weathered parent materials lacking the properties of the solum and organic materials. The depth of A-horizon soil was 10 cm on a slope just below a ridge at our study site, and the B-horizon ranged from 10–50 cm in depth.

The forest comprised deciduous broad-leaved species such as *Quercus serrata* Thunb., evergreen broad-leaved tree species such as *Ilex pedunculosa* Miquel, and some coniferous tree species. We decided to study *Q*. *serrata* and *I*. *pedunculosa* because they were the dominant species in the natural forests at the study site, representing 33% and 18% of the total aboveground biomass, respectively. The total fine root biomasses of *Q*. *serrata* and *I*. *pedunculosa* were 811.8 and 925.5 g/m^2^, respectively [26]. The two species possess contrasting types of mycorrhizal infections. The roots of *Q*. *serrata* have ectomycorrhizal fungal infections, while the roots of *I*. *pendunculosa* have arbuscular mycorrhizal fungal infections.

### Experimental setup to analyze fine root decomposition

During early May 2010, using pruning shears and a trowel, the fine root segments of *Q*. *serrata* and *I*. *pedunculosa* trees were collected from the A layer (0–5 cm) of their original forest stands. In the laboratory, large root systems were carefully isolated from the soil and organic matter and washed gently to remove soil without disrupting any of the small root tips. All living roots of *Q*. *serrata* and *I*. *pedunculosa* were sorted according to diameters into <0.5 and 0.5–2.0 mm classes. After sorting, the root segments were dried at 50°C for 48 h to avoid degradation of total phenolics [30]. Each 10 cm × 5 cm litter bag was made of nylon netting (0.85-mm mesh) and was filled with the equivalent of 0.4 g dried root samples. We established a study plot of 10 m × 30 m and divided into three subplots of 10 m × 10 m in the forest where *Q*. *serrata* and *I*. *pedunculosa* dominated. On June 27, 2010, the litter bags were inserted into 0–5 cm depth in A layer at an angle of about 45°. In total, 108 root litter bags (two species × two diameters × three replicates × nine sampling times) were tested.

In each subplot, we sampled one soil block for each root diameter class of each species sequentially on July 26, 2010 (29 days), October 2, 2010 (97 days), December 23, 2010 (179 days), March 26, 2011 (273 days), June 25, 2011 (364 days), October 1, 2011 (461 days), December 28, 2011 (549 days), April 7, 2012 (650 days), and June 17, 2012 (721 days). One litter bag was included in one soil block (width × length × depth = 15 × 10 × 10 cm). All samples were placed in sealed polyethylene bags and transported within a few hours to the laboratory for further analysis. An additional set of six litterbags per diameter class and per species was used to determine initial chemistry and ash content.

### Laboratory measurement of microbial respiration

The microbial respiration rates of root litter were measured in a temperature-controlled room at 25, 20, and 15°C using a closed dynamic chamber system equipped with an infrared gas analyzer (IRGA; LI-840, LI-COR, Lincoln, NE, USA). During measurement, the chamber lid was sealed with rubber and closed with four locks (up, down, left, and right), thereby preventing air from passing in or out. The air was circulated in a loop between the chamber and IRGA at a flow rate of 1.0 L/min using a pump (MP-15CF, Shibata, Tokyo, Japan). In the closed dynamic chamber, the root respiration rate was calculated on the basis of the slope of the linear CO_2_ concentration increase within the chamber. Short measurement time in the closed dynamic chamber system approves the accurate measurement of microbial respiration. The surface temperatures of the roots were directly measured using an infrared thermometer (AD5612A, A&D, Tokyo, Japan).

Before starting the respiration measurements, the litter bags were gently brushed free of soil and other extraneous materials, and the litter was kept for a few hours at 25°C in a temperature-controlled incubator (Eyela LTI-1000SD, Tokyo Rikakikai, Tokyo, Japan). First, the microbial decomposition respiration rate of root litter was measured in the laboratory at a fixed temperature of 25°C. The respiration rate was also measured at fixed temperatures of 20°C and 15°C, after allowing the litter samples to equilibrate to each measurement temperature for few hours. These procedures were repeated for each root sample. Between each procedure, the samples were placed in a ziploc plastic bag to prevent moisture loss from the roots while moving to the next temperature. The range of temperatures was intended to encompass the actual soil temperatures found at the site at the time of sampling. The respiration measurements were made within 12 h of sample collection, following the method as proposed by Chen et al. [20] and Kawamura et al. [21]. The temperature coefficients of the microbial respiration for each period, each species, and each diameter class were used to calculate its respiratory *Q*_10_. The *Q*_10_ was used to describe the temperature sensitivity of respiration in the measurement temperatures of 15, 20 and 25°C and was calculated on the basis of the proportional increase in microbial respiration for each 10°C rise in temperature by fitting a single-exponential model [*Q*_10_ = e^(10 × slope)^].

### Remaining mass determination

All root segments in the same samples used for respiration analyses were dried to a constant mass at 50°C and weighed. The remaining masses of the root samples were calculated on the basis of changes in the dry mass of roots using samples collected at different time intervals. A single exponential model was fitted to the remaining mass of fine roots using the least squares method, as follows:

$$Y_{t}=Y_{0}e^{-kt},$$

where *Y*_0_ is the percentage of the initial mass of fine roots, *Y*_*t*_ is the percentage of the initial mass remaining at time *t*, and *k* is the decomposition rate constant. Next, all samples were finely milled before chemical analysis. To correct for residual soil particles on the roots, each root sample was ashed in an oven for 4 h at 550°C. All mass and nutrient data for roots were expressed on an ash-free dry mass basis.

### Chemical analysis

The root samples used for the initial root analyses and decomposing root analyses at each collection point were milled for chemical analysis. For the initial root analysis, we used three replicates per diameter and per species to measure total C and N, and acid insoluble “lignin” fraction (AIF). Total C and N concentrations were measured using an NC analyzer (Sumigraph NC-900, Shimadzu, Japan). AIF concentration in the samples was estimated by gravimetry following the standard hot sulfuric acid digestion method [31].

### Data analysis

We calculated the mean values for all initial chemical properties per diameter class and species (n = 3). Two-way analysis of variance (ANOVA) was performed with diameter and species as main effects. Independent sample t-tests were performed to test for significant differences (*p* < 0.05) in the initial chemical between the diameter classes.

The microbial respiration, remaining mass, and C/N ratio for each tree species and each root diameter at every collection time point were calculated by averaging the values for the root litter samples (n = 3). *Q*_10_ was calculated for each tree species and each diameter at every collection time point using a single-exponential model based on the microbial respiration and temperature of the root samples. The remaining root mass, C/N ratio, and *Q*_10_ values for the two tree species were subjected to repeated measures ANOVA, with diameter as main effect and time as repeated factor. Percentage data for remaining mass was transformed by arcsin-square root transformation before statistical analyses for reducing heteroscedasticity in these types of data. Decomposition constants (*k*) were estimated by linear regression of ln transformed data (exponential model). Differences in *k* value were determined with analysis of covariance (ANCOVA) to compare slopes among diameter, after mass remaining were log-transformed to reduce heteroscedasticity. The relationships between *Q*_10_ and soil temperature among diameter classes and species were examined by linear regression analysis. Because the decomposition rate may vary over time, we evaluated separately the relationships between *Q*_10_ and soil temperature for the first year and for the second year. The regression slopes were compared to test whether the relationship changed significantly between first year and second year using ANCOVA. All statistical analyses were performed using the statistical package R 2.14.1.

## Results

### Initial root chemistry and morphology

In initial root chemistry, AIF, N, C:N ratio (C/N), and AIF:N ratio (AIF/N) significantly differed between two diameter classes (Table 1, Two-way ANOVA, *p* < 0.001). The <0.5 mm roots had approximately twice the N concentration of 0.5–2 mm roots (*p* < 0.001). C/N was significantly higher in 0.5–2 mm roots than in <0.5 mm roots in both species (*p* < 0.001). In both species, the <0.5 mm roots had significantly higher concentration of AIF than 0.5–2 mm roots (*p* < 0.01). The AIF/N was significantly lower in <0.5 mm roots than 0.5–2 mm roots in both species (*p* < 0.001).

**Table 1:** Initial chemical properties of fine root tissue (n = 3) in two diameter classes (<0.5 mm, 0.5–2 mm) of *Quercus serrata* and *Ilex pedunculosa* at Yamashiro Forest (mean with SE in parentheses).

Root litter	Initial root nutrients (mg g^-1^ root)		Ratios of	
	AIF	N	C/N	AIF/N
Quercus serrata				
0–0.5 mm	405.1(0.7)a	16.4(0.7)a	31.1 (1.1)a	24.7(1.2)a
0.5–2 mm	324.3(0.4)b	9.4(0.4)b	52.2 (0.8)b	34.5(0.7)b
Ilex pedunculosa				
0–0.5 mm	374.6(0.9)a	14.5(0.8)a	37.4 (1.2)a	25.8(1.4)a
0.5–2 mm	319.4(0.9)b	7.6(0.6)b	70.2 (0.9)b	42.0(1.0)a
				
p values				
Diameter	***	***	***	***
Species	*	*	**	*
Diameter × Species	**	ns	ns	ns

Means with different letters are significantly different between two diameter classes (t-test, *p* < 0.05). Abbreviation of fractions: AIF, acid insoluble fraction. Probabilities from two-way ANOVA of chemical properties between two diameter classes or the two species. Significance: *** *p* < 0.001; ** *p* < 0.01; * *p* < 0.05, ns Not significant.

### Response of microbial respiration to soil temperature

**Fig 1:**
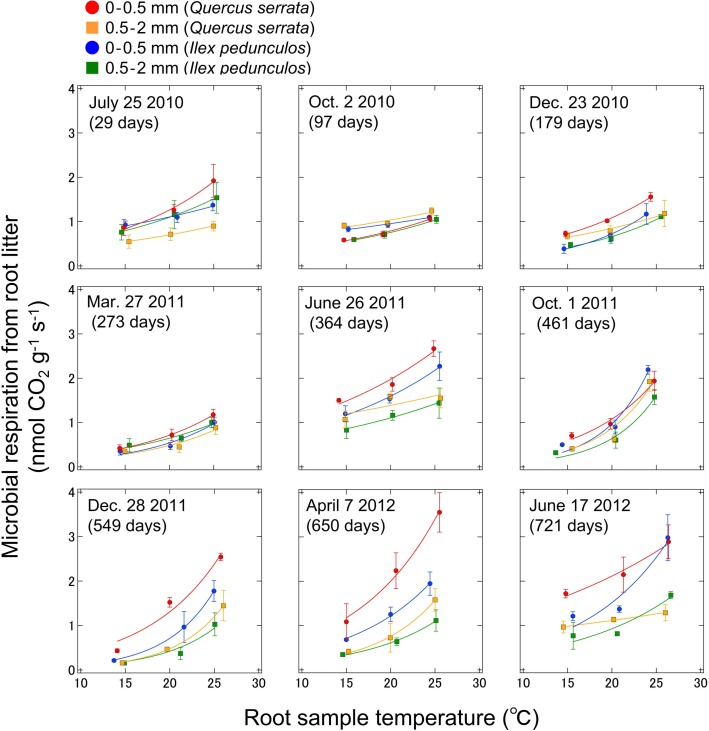
Relationship between microbial respiration and root sample temperature in the 0–0.5 and 0.5–2 mm root diameter classes of *Quercus serrata* and *Ilex pedunculosa* (at 15, 20, and 25°C) at each collection time point. The line for each period, each species, and each diameter class is fitted using a single-exponential model.

The potential microbial respiration rates were measured at fixed temperatures for each diameter class of the two species at every collection time point. The microbial respiration rates of root litter for the 0–0.5 and 0.5–2 mm root classes at the test temperatures (15–25°C) were 0.42–3.55 and 0.16–1.93 nmol CO_2_ g^−1^ s^−1^ for *Q*. *serrata* and 0.21–2.98 and 0.16–1.68 nmol CO_2_ g^−1^ s^−1^ for *I*. *pedunculosa*, respectively. For all collected samples, the microbial respiration rate of root litter tended to be higher in the <0.5 mm root class than in the 0.5–2 mm root class of *Q*. *serrata* and *I*. *pedunculosa* throughout the study period. The microbial respiration rates at each collection time point were significantly associated with changes in the constant measurement temperatures of 15, 20 and 25°C, suggesting an exponential relationship between respiration and temperature (Fig. 1).

### Seasonal patterns in the soil temperature, remaining mass, C/N ratio, and *Q*_10_ value

**Fig 2:**
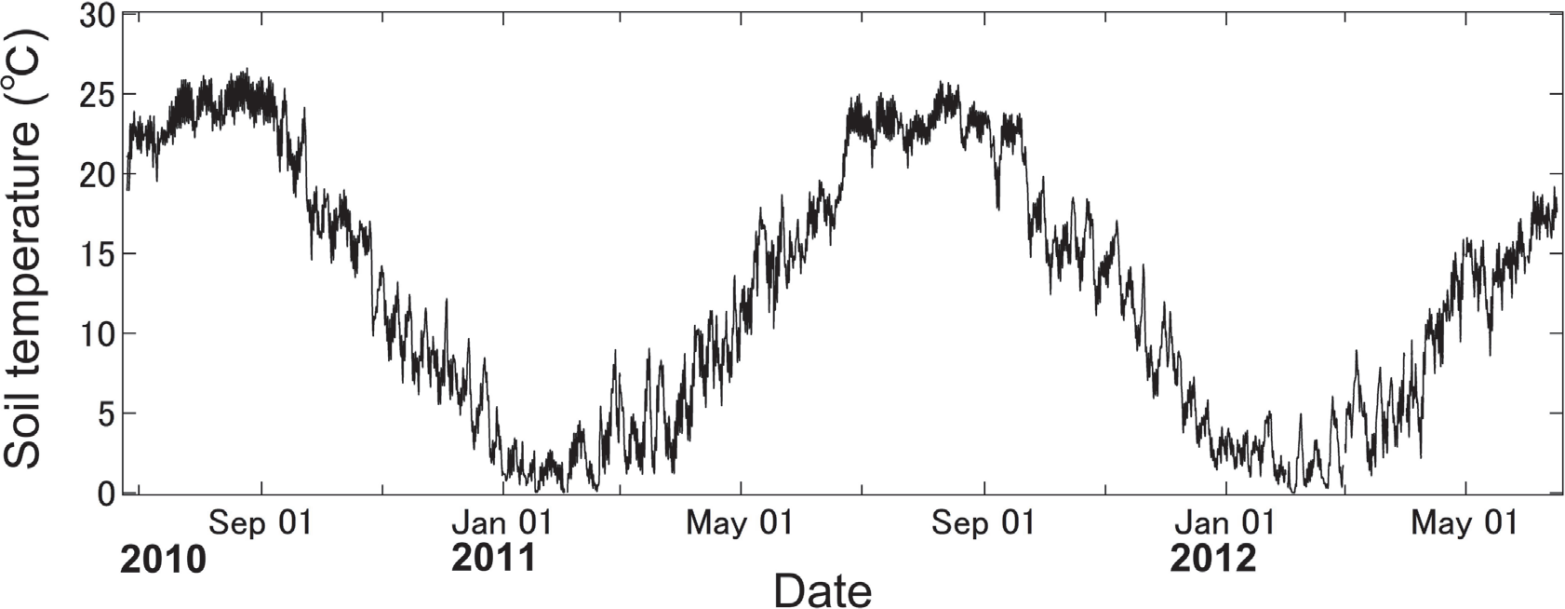
Seasonal course of soil temperature at 10 cm depth during decomposition period (July 14^th^, 2010 – July 24^th^, 2012).

The daily mean *soil temperature* at the site was lowest in February (−1.0°C), but it increased rapidly from March to July, remained high throughout the summer (<26°C) during the growing season, and decreased after mid-September (Fig. 2).

**Fig 3:**
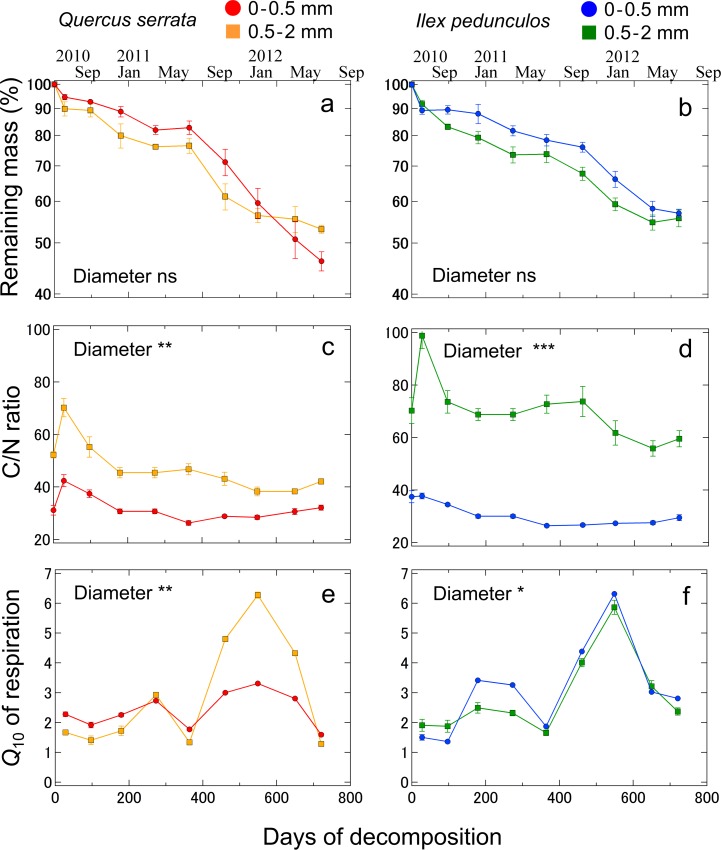
Temporal changes in (a) litter C/N ratio, (b) remaining mass, and (c) *Q*_10_ of respiration for root litter samples of the 0–0.5 and 0.5–2 mm root diameter classes of *Quercus serrata* and *Ilex pedunculosa*. Statistical significance was determined by a repeated measurements ANOVA (***, *p* < 0.001; **, *p* < 0.01; *, *p* < 0.05; NS, not significant).

As the fine root litter decomposed in both diameter classes for each species, the mass decreased over time following a quasi-exponential function (Fig. 3A and 3B). The proportion of remaining mass did not significantly change over time with root diameter (*p* > 0.05 for both species). The *k* values of the 0–0.5 and 0.5–2 mm root classes during the entire period were 0.32 and 0.35 for *Q*. *serrata* and 0.27 and 0.34 for *I*. *pedunculosa*, respectively. Although, the 0–0.5 mm root in both species tended to have lower *k* value than the 0.5–2 mm root after 2 years of decomposition, *k* value for the both species did not differ significantly between diameters (ANCOVA, *p* > 0.05).

After two years, the C/N ratios (mean ± SE) of the 0–0.5 and 0.5–2 mm root classes were 32.1 ± 0.9 and 42.1 ± 1.0 for *Q*. *serrata* and 29.5 ± 1.1 and 59.5 ± 3.1 for *I*. *pedunculosa*, respectively. The 0–0.5 mm litter root classes of both species had significantly lower C/N values than the 0.5–2 mm root classes throughout the study period (*p* < 0.01 for *Q*. *serrata*, *p* < 0.001 for *I*. *pedunculosa*; Fig. 3C and 3D), although both diameter class had similar dynamics. During the first year of decomposition, the C/N ratio of the 0–0.5 mm roots remained constant, whereas that of the 0.5–2 mm roots decreased with the decay period. During the second year, the C/N ratio of both diameters of both species remained almost constant.

The *Q*_10_ values of the microbial respiration rates of root litter for the 0–0.5 and 0.5–2 mm root classes were 1.59–3.31 and 1.28–6.27 for *Q*. *serrata* and 1.36–6.31 and 1.65–5.86 for *I*. *pedunculosa*, respectively (Fig. 3E and 3F). Changes in *Q*_10_ values were related to seasonal patterns of soil temperature; the values were higher in winter than in summer. Moreover, seasonal variations in *Q*_10_ were larger in the 2-year decomposition period than in the 1-year period. Throughout the study period, a significant difference in *Q*_10_ was observed between the diameter classes (*p* < 0.01 for *Q*. *serrata*, *p* < 0.05 for *I*. *pedunculosa*).

### Relationship between *Q*_10_ of microbial respiration and *in situ* soil temperature

**Fig 4:**
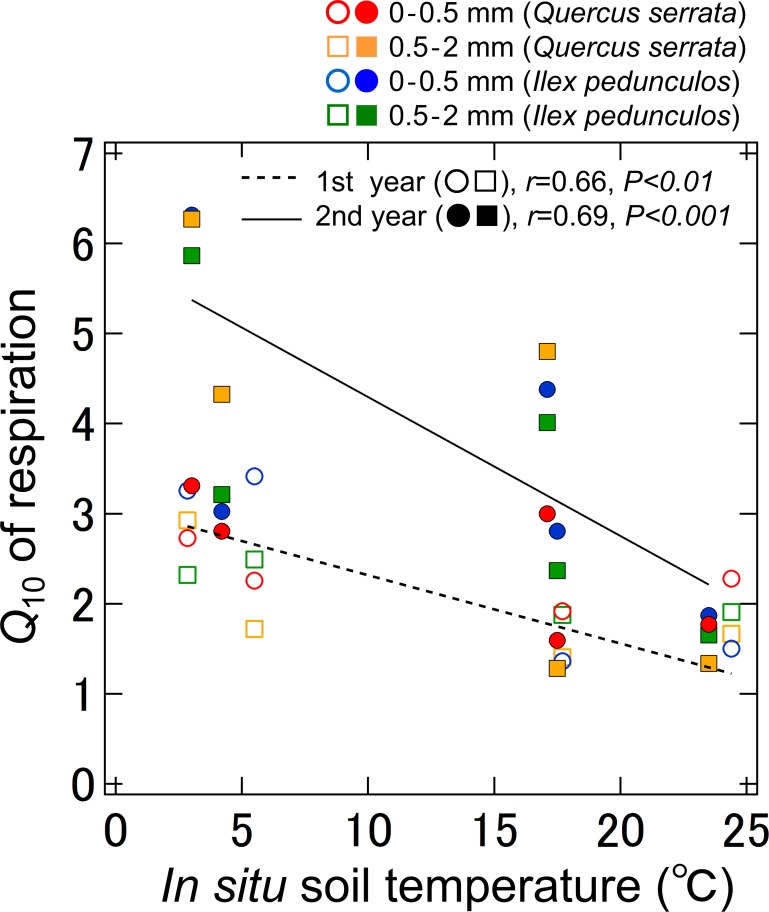
*Q*_10_ of the microbial respiration rates of root litter relative to *in situ* soil temperature in the 0–0.5 and 0.5–2 mm root diameter classes of *Quercus serrata* and *Ilex pedunculosa*. The *Q*_10_ values are means (±SEs). The values of soil temperatures show the actual soil temperatures found at the site at the time of sampling. A linear regression (dotted line, open symbols) was fitted to first year root litter data, and the temperature dependency of *Q*_10_ of microbial respiration is *Q*_10_ = −0.0702 × *T*_soil_ + 3.073. Another linear regression (solid line, closed symbols) was fitted to second year root litter data, and the temperature dependency of *Q*_10_ of microbial respiration is *Q*_10_ = −0.1942 × *T*_soil_ + 5.837. The regression were significant difference in slopes between first year and second year (ANCOVA, *p* < 0.01).

During first year of decomposition, the *Q*_10_ values of root litter declined markedly with increasing *in situ* soil temperature, irrespective of the species and diameter classes, which explained a significant proportion of the variation in the temperature sensitivity of microbial respiration in root litter (*r* = 0.66, *p* < 0.01; Fig. 4). When the second year is considered, there was a negative correlation between the *Q*_10_ values and soil temperature (*r* = 0.69, *p* < 0.001). The regressions were significant difference in slopes between first year and second year (ANCOVA, *p* < 0.01).

## Discussion

In this study, we observed temperature sensitivity during microbial respiration in dead fine root litter in two diameter classes (0–0.5 and 0.5–2.0 mm) of *Q*. *serrata* and *I*. *pedunculosa*. The *Q*_10_ values of microbial respiration rates of root litter were 1.3–6.3 at the temperatures tested (15–25°C). Previously, Chen et al. [20] showed that *Q*_10_ of litter decomposition rate of tree roots was 1.4–4.0. Boone et al. [6] and Epron et al. [3] reported that the *Q*_10_ range for microbial respiration was 2.3–2.5, which was calculated on the basis of the soil CO_2_ efflux without plant roots. Compared with the *Q*_10_ values of soil respiration, our *Q*_10_ values were well within the global median of 2.4 [1], the range (1.4–2.0) for different biomes [7], and the range (2.0–6.3) reported for European and North American forest ecosystems [4, 5].

However, *Q*_10_ values in this study showed no fixed value. The response of microbial respiration rates from root litter to various temperatures strongly differed among seasons (Fig. 1). Furthermore, there were seasonal patterns in the *Q*_10_ values of microbial respiration in both species and diameter classes with temperature; *Q*_10_ was highest in winter and lowest in summer, even when compared at the same measurement temperatures (Fig. 3E and 3F). The seasonal patterns in temperature sensitivity in our study are consistent with those reported by Kirschbaum [32] and Hamdi et al. [33], who showed that with increasing temperature, there is a declining relative increase in the fraction of molecules with sufficient energy to react, and consequently, the *Q*_10_ value decreases.

These seasonal patterns in temperature sensitivity might have been related to microbial physiological acclimation and community adjustment. It is known that microorganisms at low temperatures induce a suite of cold acclimation mechanisms by changing their lipid composition, synthesizing new proteins, and changing their resource allocation from growth to survival mechanisms [34, 35]. Allison et al. [36] reported that the microbial response to low temperature was enhanced by the size of the microbial community, enzymatic degradation potential, and C mineralization rate. In fact, temperature could directly or indirectly affect the microbial growth [37], the biomass and diversity [38], and the overall metabolism of the microorganisms, including production of degradative enzyme [39]. Thus, low temperature acclimation and addaptation of microorganisms may lead to higher temperature sensitivity of microbial respiration. Furthermore, when soil water content decreases, the metabolic activity of most microbial species decreases, resulting in decreased temperature sensitivity and nutrient mineralization [12, 34]. However, most of works on the temperature sensitivity of litter decomposition have been based on laboratory conditions. Future research on forest field experiments needs to consider more explicitly soil temperature effects on the degree of microbial acclimation, community adjustment and water status, thereby leading to an increase and decrease in the temperature sensitivity of microbial respiration after acclimation to colder and warmer temperatures, respectively [40].

The analysis of the litter quality of dead fine roots during the decay period also has important implications for temperature sensitivity. We found that the *Q*_10_ values of both root diameter classes of the two species were related to the seasonal soil temperature pattern, but the variations in these values were larger in the second year of decomposition than in the first year (Fig. 3E and 3F). In addition, the relationship between *Q*_10_ and soil temperature exhibited significant difference in slopes between first year and second year (Fig. 4). These findings suggest that substrate quality and temporal differences in substrate availability contribute to the large variability in the *Q*_10_ value. Recent studies of SOM reported that the temperature sensitivity of decomposition is higher with recalcitrant substrates than with labile compounds [13, 14]. The high activation energy required for the decomposition of recalcitrant substrates might lead to a higher temperature sensitivity of decomposition. Fierer et al. [17] showed that the temperature sensitivity of leaf litter decomposition increased as the litter quality decreased during decay. Similarly, as the fine root litter of both diameter classes and species decomposed throughout the two years of this study, its mass also decreased approximately over time (Fig. 3A and 3B). The C/N ratio of the roots also increased at 1 month, decreased after 1 year, and then remained constant up to 2 years (Fig. 3C and 3D). Thus, the proportion of the overall root litter that comprised degradable compounds (i.e., labile and recalcitrant) may vary during the decay period, which could control the temperature sensitivity of root decomposition, where low quality litter may be more sensitive. However, this is still debated for root litter where both litter quality and other physicochemical mechanisms may regulate degradability [41].

Furthermore, the microbial respiration rate and *Q*_10_ values of both root diameter classes of the two species had similar seasonal patterns, but the magnitudes of changes differed between the diameter classes (Fig. 3E and 3F). This suggests that the microbial physiological performance may have been higher in the 0–0.5 mm roots than in the 0.5–2 mm roots, thereby leading to variation in the decomposition process within the fine roots and in the temperature-sensitive responses of microorganisms. Recent studies of fine root decomposition have detected differences in the decomposition rate and nutrient dynamics among diameter classes, thereby demonstrating the effect of root litter quality on decomposition [27, 29]. Living fine roots comprise multiple branching orders that correspond to the root diameter, which vary widely in their chemical and physical properties because of their different physiological functions [25, 26]. In the present study, the <0.5 mm roots had significantly higher concentration of N and AIF than 0.5–2 mm roots (Table 1). Furthermore, the initial roots of <0.5 mm had a lower C/N ratio and AIF/N ratio than the 0.5–2 mm roots. Because lignin is considered more stable component [23, 24], high AIF content of <0.5 mm in our study would result in the different decomposition process due to lower energy supply from residual matters to microbial decomposers [28]. Thus, the root diameter represents the initial substrate quality, which may determine temperature sensitivity by affecting the magnitude of microbial respiration. However, despite these differences in *Q*_10_ between diameter class, the patterns of decomposition rates did not vary between <0.5 mm and 0.5–2 mm roots (Fig. 3A and 3B). This finding implies that there is a diameter-specific decomposition process through a combination of biological as well as physical-chemical processes [22, 23]. Determining how the changes in *Q*_10_ between fine root diameter class under different temperature will be elucidated in changes in microbial respiration and decomposition rates remains a significant challenge.

Indeed, our measurements imply some technical problems in the methodology for evaluation of microbial respiration and *Q*_10_. For example, when calculating *Q*_10_ values among measurement temperatures, the physical disturbance by altering temperatures may create artificially enhanced respiration rates. As result, the basal respiration rate of a litter sample cannot directly be scaled up to stand-level fluxes. Nevertheless, it shows a physiological potential response of the soil microorganisms to temperatures. Future advances in techniques, with minimizing the disturbance of natural conditions, will elucidate the accurate response of fine root litters to temperatures, allowing us to quantify the soil C sequestration and the C fluxes driven by decomposition processes in forest ecosystems.

Overall, we detected strong relationships between the *Q*_10_ value and *in situ* soil temperature during the different stages of decomposition in different diameter classes (Fig. 4). We also found a large variation in the temperature sensitivity of microbial respiration rates of root litter even during the early stage of decomposition. These results support our hypothesis that the temperature sensitivity of microbial respiration in root litter differs with the decay period and season. To our knowledge, this is the first study to examine the short-term temperature-sensitive responses of microorganisms using fine root litter less than 2 mm in a natural forest. The temperature sensitivity of fine root decomposition is an element of uncertainty, but it has major consequences in the current models of C turnover in soils. Therefore, it is a subject of great debate in the context of global climate change [12]. Future increases in temperature could generate a positive feedback between global warming and soil C flux along the stage of litter decomposition. Most empirical models of soil C dynamics may over- or underestimate the soil C flux and soil C sequestration because they depend on the temperature responses of soil respiration or SOM fractions and fail to consider temperature sensitivity during the early stage of litter decomposition [42]. We consider that advances in our understanding of the temperature sensitivity of microbial decomposition of tree fine roots will provide a breakthrough in our ability to understand the processes of litter decomposition and the prediction of soil C dynamics in a future climate.
